# The Advancement in Detecting Sepsis and Its Outcome: Usefulness of Procalcitonin in Diagnosing Sepsis and Predicting Fatal Outcomes in Patients Admitted to Intensive Care Unit

**DOI:** 10.7759/cureus.14439

**Published:** 2021-04-12

**Authors:** Mushrin Malik, Archana Sreekantan Nair, Janan Illango, Nabeel Siddiqui, Rajvi Gor, Ransirini W Fernando, Pousette Hamid

**Affiliations:** 1 Research, California Institute of Behavioral Neurosciences & Psychology, Fairfield, USA; 2 Neurology, California Institute of Behavioral Neurosciences & Psychology, Fairfield, USA

**Keywords:** sepsis, procalcitonin, diagnosis & prognosis, sirs, inflammatory biomarker

## Abstract

Sepsis still remains a big challenge in patients admitted to intensive care units (ICUs) despite stellar advances made in the field of medicine. We can achieve better clinical outcomes in patients by diagnosing sepsis earlier. Procalcitonin (PCT), an inflammatory biomarker, has shown promising results in this regard. Therefore, this systematic review was done to assess the use of PCT in diagnosing and predicting severe outcomes in patients admitted to ICU and to assess if introducing PCT as a routine biochemical tool in hospitals would be helpful to achieve better clinical course in ICU patients.

To identify relevant articles, we searched PubMed, Google Scholar, and references of included articles. Eligible studies were identified by two investigators independently and data were extracted. Original articles that evaluated the diagnostic and prognostic value of serum PCT levels in predicting sepsis, the severity of sepsis, and mortality among adult patients admitted to ICU were included in this study. A total of 2,063 citations were identified by the search, among which 10 studies (five prospective cohort, three retrospective cohort, one cross-sectional, and one case-control study) met the inclusion criteria. Most studies showed moderate-to-low risk of bias which was evaluated using the Quality in Prognosis Studies tool. All studies showed a positive correlation between initial PCT levels and detecting mortality resulting from sepsis, six studies found PCT helpful in detecting sepsis, and four studies evaluated the role of PCT in detecting severity in patients with sepsis. One study found area under the curve of serum PCT level for predicting 28-day mortality to be 0.82 (95% confidence interval [CI]: 0.70-0.94; p < 0.001) in adults and 0.83 (95% CI: 0.73-0.92; p < 0.001) in the elderly having an optimal cut-off level of serum PCT of 0.2 ng/mL in both the adult and elderly groups, with a sensitivity of 81 and 75% and specificity of 81.7 and 80.4%, respectively.

PCT has shown promising results in detecting sepsis and its clinical course. For early diagnosis and management of sepsis, severe sepsis, and mortality in patients admitted to the ICU for a more favorable clinical outcome, PCT can be used.

## Introduction and background

**Figure 1:**
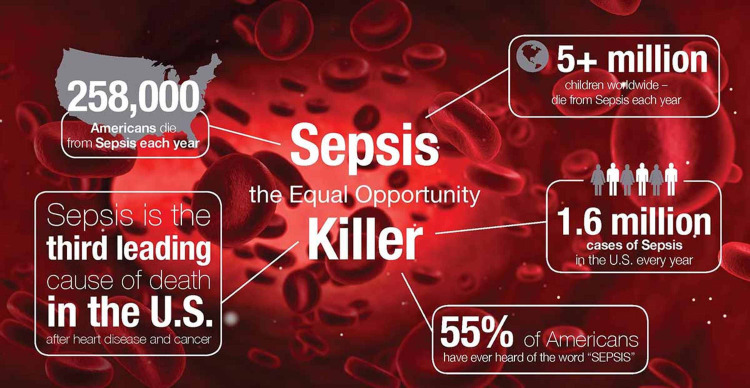
Sepsis: the silent killer.

Despite pathbreaking advancements in the medical field, the discovery of more refined management protocols, and extensive coverage of antimicrobials, sepsis remains as one of the leading causes of death worldwide. According to the World Health Organization, sepsis kills 11 million people each year and disables millions more. According to the Centers for Disease Control and Prevention, at least 1.7 million adults in the United States are affected by sepsis each year, of whom nearly 270,000 Americans die, as shown in Figure 1.

Systemic inflammatory response syndrome (SIRS) is defined as fever (>38°C) or hypothermia (<36°C); increased heart rate (>90 beats/minute), tachypnea (>20 breaths/minute), or hyperventilation (PaCO_2_ < 32 mmHg); and altered white blood cell count (WBC) (>12,000 cells/mm^3^ or <4,000 cells/mm^3^) or presence of >10% immature neutrophils. SIRS resulting from infection is defined as sepsis. Sepsis is severe when it presents with at least one major organ dysfunction, hypotension, or hypoperfusion [1,2]. Due to the lack of specific clinical signs, it is difficult to identify patients with sepsis. Moreover, because sepsis is defined as SIRS along with the source of infection, a large number of patients can be misdiagnosed with sepsis without having one [1]. On the other hand, the progression of SIRS to sepsis to severe sepsis can occur rapidly in some patients resulting in fatal complications [1]. Therefore, it is very important to diagnose sepsis at an early stage with diagnostic tools having high sensitivity and specificity.

Although microbiological cultures remain the gold standard for diagnosis of sepsis, they are time-consuming and might not always mirror the host immune response, as well as might not be positive in patients receiving antimicrobials. Inflammatory biomarkers, such as C-reactive protein (CRP), erythrocyte sedimentation rate (ESR), and WBC count, show poor sensitivity and specificity. However, procalcitonin (PCT), a biomarker, has garnered much attention worldwide for its higher accuracy in diagnosing and monitoring sepsis [3].

In a state of systemic inflammation, PCT, a prohormone of calcitonin is secreted by many cell types in response to bacterial endotoxins [4]. Higher the endotoxin level, higher is the PCT production; therefore, a PCT level of >0.1 ng/mL indicates a bacterial infection, and a PCT level of >0.5 ng/mL indicates severe sepsis [5-7]. PCT level starts to rise within 2-4 hours of systemic inflammation and peaks at 8-24 hours with a half-life of 24 hours, making diagnosis faster and monitoring disease progression better than other conventional inflammatory biomarkers such as CRP (rises within 12-24 hours of systemic inflammation and stays elevated for 3-7 days) [8].

Therefore, PCT can be regarded as a promising biomarker in the diagnosis, prognosis, and monitoring of patients with systemic inflammation. This systematic review is an attempt to shed light on how accurate, if at all, is PCT measurement in diagnosing and predicting the prognosis of patients with sepsis for a more favorable outcome, and if measuring PCT routinely in the intensive care unit (ICU) setting would be beneficial.

## Review

Method

The study selection, data extraction, and methodological quality assessment steps described below were adopted with modification from AlRawahi et al. [9].

Search Strategy

According to the Preferred Reporting Items for Systematic Reviews and Meta-analyses [10] guidelines, a systematic literature review was performed using databases starting from January 2021. The databases included PubMed and Google Scholar. The following Medical Subject Heading Terms and keywords were used: Procalcitonin, Diagnosis & Prognosis, Sepsis, SIRS, and Inflammatory Biomarkers. The search only included original studies on human subjects published in the English language. To include pertinent papers, the “related articles” feature from PubMed was used, and two authors independently searched for additional citations from the reference list of included articles.

Study Selection

The screening process to recognize all citations of potential acceptability was performed by two reviewers (M.M. and A.N.) independently. The inclusion criteria included (1) study participants (adults ≥18 years of age), (2) intervention (single or serial measurements of serum PCT level from the day of the admission to ICU admission), (3) comparison of the prognostic performance of PCT levels compared to other inflammatory biomarkers or no intervention, (4) outcome (documentation of at least one of the outcomes of sepsis, mortality, or correlation of PCT with the severity of infection), and (5) cohort, case-control, or cross-sectional study designs.

For final eligibility, full-text papers of recognized abstracts that were pertinent to our inclusion criteria were evaluated. Case reports, letters, conference abstracts, and editorials were excluded. The inter-rater reliability of the two reviewers was evaluated using Kappa statistic [11].

Data Extraction

Data were extracted independently by two reviewers (M.M and A.N.) using a standardized recording tool to document the study design and setting, year of publication, country of origin, number of study participants, clinical characteristics of participants, PCT testing system, kinetics of PCT, and study outcomes.

The terms “SIRS” and “sepsis” were defined according to the American College of Chest Physician/Society for Critical Care Medicine [1]. As most studies were published prior to the release of Sepsis-3, the definition of SIRS, sepsis, and severe sepsis were supported [12].

Methodological Quality Assessment

Two independent investigators (M.M. and A.N.) evaluated the risk of bias of the recognized studies using the Quality in Prognosis Studies tool developed by Hayden et al. [13]. This tool includes 30 criteria divided into six domains: patient selection, study attrition, prognostic factor measurement, outcome measurement, confounding measurement and account, and statistical analysis and reporting. Each criterion was scored as “yes,” “no,” or “unclear.” Therefore, each domain was judged as being of “low,” “moderate,” or “high” risk of bias according to the scoring system. When the bias was rated as low or moderate with respect to almost all of the domains, a study was considered to be of high quality. On the contrary, when the bias was rated high in most of the bias domains, a study was considered to be of low quality. Consensus resolved any disagreements.

Results

Literature Search

**Figure 2:**
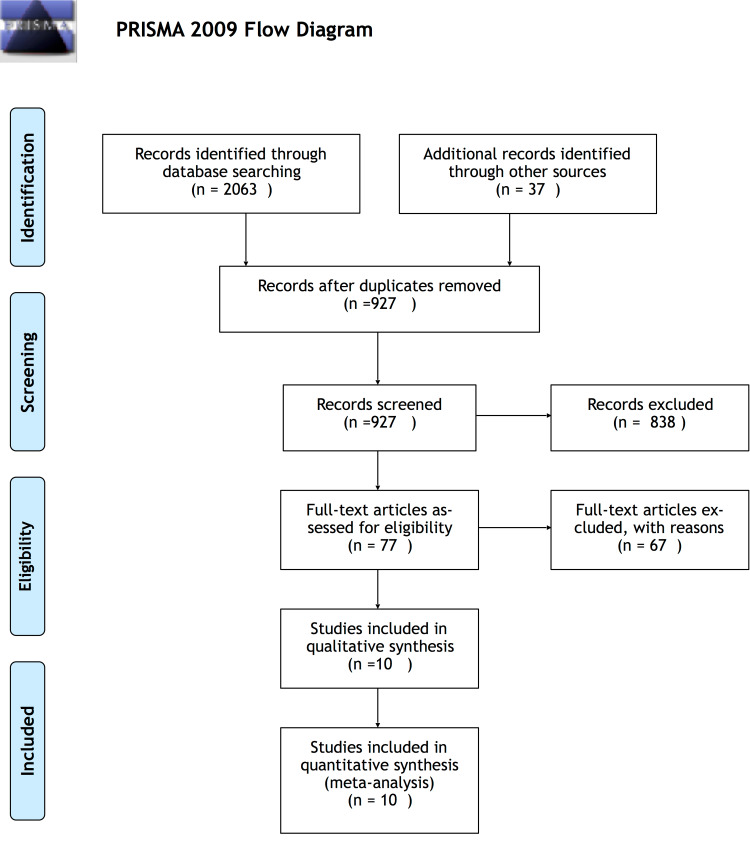
PRISMA flow diagram. PRISMA, Preferred Reporting Items for Systematic Reviews and Meta-analyses

A flow diagram of study identification and subsequent inclusion is shown in Figure 2. A total of 2,063 citations were identified by the search, among which 10 studies were included in this systematic review due to their high quality. A total of 4,018 patients admitted to the ICU were included in the study.

Characteristics of the Included Studies

The characteristics of the included studies are shown in Table 1. The studies included in this review were published from 2009 to 2019, with most of the studies being published after 2014 (90%). The following 10 studies were included in the review: Nargis et al. [14], Mustafić et al. [15], Cui et al. [16], Lipińska-Gediga et al. [17], Shokouhi et al. [18], Ryoo et al. [19], Peschanski et al. [20], Demirdal et al. [21], Tsangaris et al. [22], and Yu et al. [23]. Among the 10 studies, six were conducted in Asia [16,18,20,21,23] and four were conducted in Europe [15,17,20,22]. We only included observational and non-interventional studies. Five of these studies were prospective cohort [15,17,19,21], three studies were retrospective cohort [16,20,23], one study was case-control [17], and one study was cross-sectional [13]. The role of PCT in diagnosing and predicting the prognosis of at least one clinical outcome (sepsis, severe sepsis, or death) in patients admitted in the ICU was evaluated. A total of 4,018 patients admitted in the ICU were included in this study to evaluate the role of PCT in predicting outcomes in patients with infectious systemic inflammation.

**Table 1:** Study characteristics. PCT, procalcitonin; ICU, intensive care unit

Study	Study design	Study setting	Number of patients	Sensitivity of PCT in detecting outcomes	Specificity of PCT in detecting outcomes	PCT detecting outcomes		
						Sepsis	Severe sepsis/ Septic shock	Death
Nargis et al. [14] (Bangladesh, 2014)	Cross-sectional	ICU	73	76.36%	72.2%	Yes	-	Yes
Mustafić et al. [15] (Bosnia & Herzegovina, 2018)	Prospective cohort	ICU	106	-	-	Yes	Yes	Yes
Cui et al. [16] (Hebei, 2019)	Retrospective cohort	ICU	59	-	-	-	-	Yes
Lipińska-Gediga et al. [17] (Poland, 2016)	Prospective cohort	ICU	50	62%	33%	-	Yes	Yes
Shokouhi et al. [18] (Iran, 2017)	Case control	ICU	176	78%	80.05%	Yes	-	Yes
Ryoo et al. [19] (Korea, 2019)	Multicenter, prospective observational	ICU	1772	-	-	-	-	Yes
Peschanski et al. [20] (Paris & Rowen, 2016)	Retrospective cohort	ICU	188	54%	72%	-	Yes	Yes
Demirdal et al. [21] (India, 2018)	Prospective cohort	ICU	226	56.8%	94.1%	Yes	Yes	Yes
Tsangaris et al. [22] (Greece, 2009)	Observational cohort	ICU	50	70%	91%	Yes	-	Yes
Yu et al. [23] (China, 2019)	Multicenter retrospective cohort	ICU	1318	-	-	Yes	-	Yes

Risk of Bias Assessment

**Figure 3:**
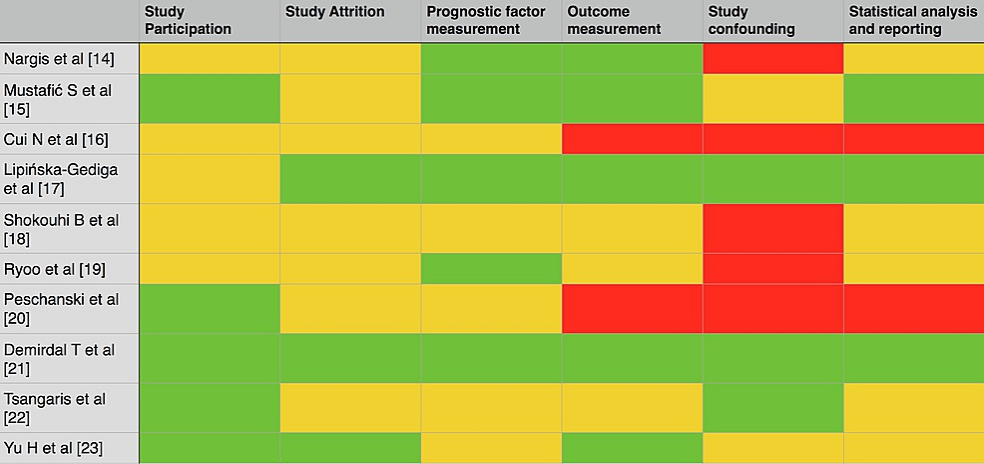
Risk of bias assessment. QUIPS, Quality in Prognostic Studies Red, high risk of bias; Yellow, moderate risk of bias; Green, low risk of bias

Figure 3 demonstrates the risk of bias assessment. Most studies had low-to-moderate risk of bias. A high risk of bias in at least one domain was found in six out of ten studies [14,16,18-20,22]. Moderate-to-high risk of bias in study confounding bias was also found in six out of ten studies [14,16,18-20,22]. Most of the studies did not have many potential confounding factors in the study design, and the effects of confounders in the analysis were adjusted, while 10% of the studies did not name any confounders [19]. In the domain of statistical analysis, six out of ten studies had a moderate-to-high risk of bias (70%) [14,16,18-20,22,23]. To assess prognostic relationships, all 10 studies used statistical models.

Kinetics of Procalcitonin

**Figure 4:**
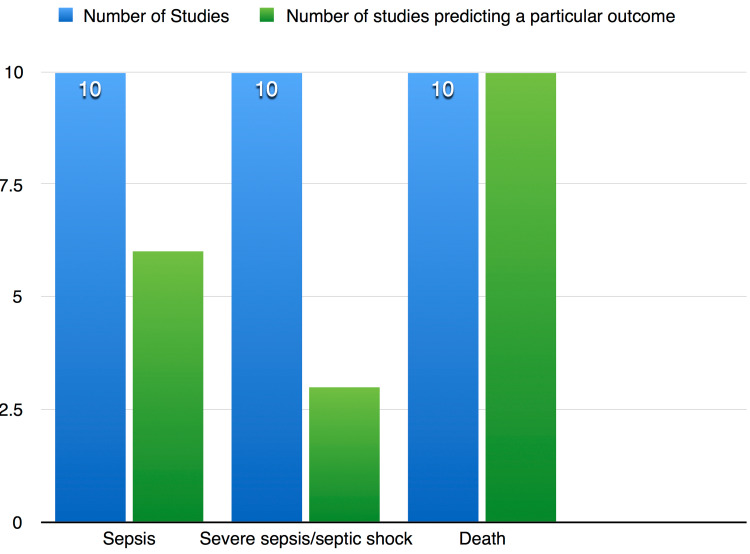
PCT in detecting outcomes in septic patients. PCT, procalcitonin

A serum sample was used to measure PCT levels in all studies. Two studies used Kryptor assay [19,21], two studies used the VIDAS system (bioMérieux, Marcy-l'Étoile, France) (20%) [15,23], one study used immunoluminometric assay [19], one study used enzyme-linked fluorescent immunoassay [14], one study used electrochemiluminescence analyzer (Cobas, Roche, Basel, Switzerland) [16], one study used enzyme-linked immunosorbent assay (Elecsys 2010, Roche, Basel, Switzerland) [18], and two studies did not mention the laboratory technique used to measure PCT [20,22]. PCT levels started to rise within 24 hours of ICU admission, with higher levels corresponding to more severe sepsis. Higher PCT levels were also associated with death in patients (Figure 4).

Procalcitonin in Predicting Sepsis in Patients Admitted to the Intensive Care Unit

A total of six out of ten studies [14,15,18,21-23] evaluated the role of PCT in detecting sepsis in patients admitted to the ICU. Nargis et al. [14] showed that the mean PCT in culture-positive patients was 10.9 ng/mL compared to a mean PCT of 7.1 ng/mL in culture-negative patients. The study showed a significantly higher value of PCT in patients with sepsis compared to patients with SIRS with a sensitivity of 76.36%, a specificity of 72.2%, and an accuracy of 75.34%. Mustafić et al. [15] found a higher mean PCT in septic patients (3.495 ng/mL) compared to patients with SIRS who had a mean PCT of 0.25 ng/mL. PCT was shown to have the best predictive value in diagnosing sepsis with a cut-off value of 0.57 ng/mL having a positive predictive value (PPV) of 98.76%, negative predictive value (NPV) of 92%, and accuracy of 97.1%. Shokouhi et al. [18] also found a positive correlation between PCT level and predicting sepsis. The area under the curve (AUC) of serum PCT level for predicting bloodstream infection was 0.81 (95% confidence interval [CI]: 0.71-0.91; p < 0.001) in the adult group and 0.73 (95% CI: 0.63-0.84; p < 0.001) in the elderly group, with an optimal cut-off level of serum PCT being 0.09 ng/mL (sensitivity, 82.6%; specificity, 82.0%) in the adult group and 0.08 ng/mL (sensitivity, 69.1%; specificity, 70.0%) in the elderly group. Demirdal et al. [21] found a statistically significant difference in PCT levels between patients suffering from SIRS and those suffering from sepsis. Mean PCT in SIRS was 0.9 ± 0.8 ng/mL, and mean PCT in septic patients was 4.2 ± 6.9 ng/mL, with a cut-off value of 0.2 ng/mL having a sensitivity of 56.8% and specificity of 94.1%. Tsangaris et al. [22] showed a significantly higher PCT level in patients with sepsis having a sensitivity of 96% and specificity of 100%. Yu et al. [23] found that increased PCT level along with quick Sepsis-Related Organ Failure Assessment (qSOFA) score of >2 could better predict the diagnosis of sepsis compared to qSOFA score alone in predicting sepsis. Using the qSOFA+PCT score, 752 (53.1%) patients could be diagnosed with sepsis compared to 208 (15.8%) patients using the qSOFA score alone.

The most common source of infection that resulted in sepsis was lung [14,15,17-22], followed by the urinary tract [14,15,18-21], gastrointestinal tract [15,17,19,21,22], and blood stream [14,22]. Two studies [16,23] did not mention the exact source of infection.

Procalcitonin in Predicting Severe Sepsis/Septic Shock in Patients Admitted to the Intensive Care Unit

A total of four studies [15,17,20,21] evaluated the role of PCT in predicting severe sepsis. Mustafić et al. [15] showed increased PCT level with increasing severity of sepsis (r = 0.95; p < 0.0001). Lipińska-Gediga et al. [17] using receiver operating characteristic (ROC) curve analysis of the septic shock diagnosis on the day of the admission found that the cut-off value for PCT was 8.01 µg/L, with an AUC of 0.72, sensitivity of 70%, and specificity of 26%. Demirdal et al. [21] showed that PCT could predict severe sepsis with a value of 14.2 ± 22.4 ng/mL in patients with severe sepsis compared to a PCT level of 4.2 ± 6.9 ng/mL in patients with only sepsis having a cut-off value of 0.2 ng/m:. However, Peschanski et al. [20] showed that a PCT value of >32.5 µg/mL could not statistically differentiate between sepsis and severe sepsis.

Procalcitonin in Predicting Mortality in Patients Admitted to the Intensive Care Unit

All 10 [14-23] studies evaluated the role of PCT in predicting mortality in ICU admitted patients. Nargis et al. [14] found a PCT level of >10 ng/mL to be associated with mortality of 16.6%. Mustafić et al. [15] found that PCT could predict 28-day mortality with a cut-off value of 15.05 ng/mL. Cui et al. [16] found that regarding changes in serum PCT level, levels in the non-survivor group on the second, third, and fifth days (34.66, 19.36,15.46 ng/mL, respectively) were higher than those in the survivor group (2.64, 1.22, 0.66 ng/mL, respectively). Lipińska-Gediga et al. [17] also showed a statistically significant higher PCT level in non-survivors with first-day PCT being 7.38 ng/mL in survivors compared to a PCT level of 11.1 ng/mL in non-survivors. Shokouhi et al. [18] found that the AUC of serum PCT level for predicting 28-day mortality was 0.82 (95% CI: 0.70-0.94; p < 0.001) in adults and 0.83 (95% CI: 0.73-0.92; p < 0.001) in the elderly with an optimal cut-off level of serum PCT of 0.2 ng/mL in both the adult and elderly groups, with a sensitivity of 81 and 75% and specificity of 81.7 and 80.4%, respectively. Ryoo et al. [19] found the 28-day mortality rate of an elevated PCT level with a cut-off value of 17 ng/mL to be 17.8%. Peschanski et al. [20] showed the mean PCT level among non-survivors to be 34 µg/mL compared to a mean PCT level of 6.4 µg/mL among survivors having a sensitivity of 54%, a specificity of 72%, PPV of 70%, and NPV of 57%. Demirdal et al. [21] found the overall mortality to be higher in patients with severe sepsis compared to patients with SIRS or sepsis. The mortality in patients with SIRS was 7.4%, sepsis was 30.8%, and severe sepsis was 61.7%. However, there was no statistical significance in predicting 28-day mortality. Tsangaris et al. [22] showed a median (interquartile range) PCT concentrations in the group of patients who survived at 28 days to be 0.28 ng/mL (0.80) and in patients who did not survive to be 1.07 ng/mL (3.09) (p = 0.004). Median PCT concentrations sequentially measured on days one, two, three, and four tended to increase in non-survivors contrary to survivors (p = 0.028). Specifically, a concentration of PCT of less than 0.5 ng/mL on the third day after the advent of fever was associated with a favorable survival. Yu et al. [23] found that the incorporation of PCT into the qSOFA score could better predict the 30-day mortality with a sensitivity of 90.9% and a specificity of 50.3%.

Performance of Procalcitonin in Diagnosis and Prognosis of Patients With Sepsis Compared to Other Biomarkers

A total of seven studies [14-17,20,22,23] evaluated the performance of other inflammatory biomarkers. Nargis et al. [14] showed CPR level to increase in sepsis compared to severe sepsis with the highest sensitivity in predicting mortality, although PCT showed the highest accuracy. Mustafić et al. [15] showed a SOFA score of more than seven to be associated with mortality, and Acute Physiology and Chronic Health Evaluation (APACHE II) score of less than nine to be associated with lower mortality and 10-19 with higher mortality. It also showed a positive correlation with raised PCT, CRP, lactate, SOFA score, and APACHE II score with a fatal outcome. Serum lactate showed the best predictive value in 28-day mortality. Cui et al. [16] found CRP level to be higher in non-survivors and patients with septic shock compared to survivors and patients with sepsis. Lipińska-Gediga et al. [17] found a higher SOFA score in patients with severe sepsis. WBC level was not statistically significant in diagnosing or predicting the prognosis of sepsis. CRP level was also not statistically significant except for the fifth-day CRP level between survivors and non-survivors. Peschanski et al. [20] showed a better predictive value of mortality of PCT with lactate compared to PCT alone, with ROC curve analysis indicating an AUC of 0.692 (95% CI: 0.594-0.780), although sensitivity was 50% and specificity was 96%, which was similar to PCT alone. Tsangaris et al. [22] showed the area under the ROC curve (95% CI) of PCT to be 0.85, WBC to be 0.65, and CRP to be 0.68. Yu et al. [23] showed the qSOFA score to be a great predictor of sepsis and mortality; however, with the combination of PCT with qSOFA score, the prognosis prediction of sepsis was significantly improved compared to the qSOFA score combined with WBC or CRP.

Discussion

In 1993, Assicot et al. described PCT as a marker of diagnosis and prognosis of systemic inflammation resulting from microbial infections [24]. PCT has since been evaluated thoroughly as a marker of systemic inflammation, sepsis, severe sepsis, and death related to sepsis. PCT has been evaluated both singularly and in combination with other markers such as CRP, qSOFA score, and WBC in adults in the ICU setup. Monitoring PCT levels has shown effectiveness in applying therapy in everyday clinical use for a better clinical outcome of a septic patient. The effectiveness of PCT in assessing sepsis and its prognosis is well established with multiple studies evaluating it. Therefore, this review will help us to have a better comprehensive understanding of the role of PCT as an inflammatory biomarker.

We found in our review that six out of ten studies evaluated the role of PCT in diagnosing sepsis in patients admitted to the ICU. All six studies showed a positive correlation between PCT and diagnosis of sepsis. The lack of consistency in defining sepsis and lack of consensus gold standard for defining infection can be the reason behind the variability in the results among studies. Therefore, patients may have been misclassified as having systemic inflammation who did not exhibit clinical signs or in whom bacterial cultures were negative. Five out of these six studies compared PCT to other biomarkers and showed PCT to have a higher sensitivity, specificity, and accuracy in diagnosing sepsis. Only one study, evaluated the role of PCT alone with a sensitivity of 56.8% and specificity of 94.1% in diagnosing sepsis [21]. We must also remember that not every patient with an infection is septic. However, studies have shown a significantly higher PCT level in patients with sepsis than those with an isolated infection, and thereby helping us identify vulnerable patients requiring more extensive management [25].

A total of three studies found PCT to effectively diagnose severe sepsis/septic shock. A significantly higher level of PCT was found in patients with severe sepsis making PCT a promising biomarker in detecting patients with poor outcomes. One study found PCT unable to statistically differentiate sepsis from severe sepsis [20].

With sepsis still being one of the leading causes of death in the world, this review aimed at assessing the role of PCT in predicting fatal outcomes in septic patients admitted to the ICU. All 10 studies evaluated the role of PCT in predicting mortality, and all the studies found a positive correlation between PCT and its ability in detecting mortality. Mustafić et al. [15] found that PCT could predict 28-day mortality with a cut-off value of 15.05 ng/mL, but serum lactate showed the best predictive value in 28-day mortality. Tsangaris et al. [22] showed a sequential rise in PCT levels measured on days one, two, three, and four in non-survivors compared to survivors (p = 0.028). Therefore, the significantly higher PCT level can help us estimate which patients are more vulnerable and need more care for a better clinical outcome. However, there were differences in time frames in defining mortality across studies; some studies used overall ICU mortality, while others used 28-day mortality. This factor might act as a limitation in assessing PCT as a biomarker of mortality in septic patients. Nevertheless, it is absolutely clear that PCT levels play a role in diagnosing sepsis, and sepsis is clearly associated with high mortality.

This systematic review attempts to comprehensively assess the diagnostic and prognostic value of PCT level to detect three outcomes, i.e., development of sepsis, severe sepsis/septic shock, and mortality in septic patients. In our review, most of the studies were prospective, which helped in the evaluation of the kinetics of PCT level from day zero of ICU admission, as well as its relationship with the development of complications later in the clinical course, thus precisely evaluating the role of PCT as an effective biomarker in septic patients.

PCT production can also be induced by non-infectious causes of systemic inflammation, such as shock, trauma, surgery, burn injury, and chronic kidney disease. However, it is observed that the rise is not as significant as the elevation of PCT level in sepsis [26,27]. However, most of our studies excluded severely debilitated/immunosuppressed patients in whom PCT level would have been raised due to non-infectious systemic inflammation. Diagnosing and predicting the prognosis of sepsis in these patients is important which has not been assessed in any of the studies we reviewed. This raises an important question of how useful is rising PCT level in determining sepsis in severely debilitated individuals and those with non-infectious causes of systemic inflammation.

Our review has multiple limitations such as not including non-translated, non-English publications. Due to the presence of confounding variables, the quality of the primary studies varied. Furthermore, different studies used different statistical methods in assessing outcomes, which made integrating results difficult, which represents a limitation of this review.

## Conclusions

All the studies included in this review found PCT to be a promising and superior biomarker in diagnosing and predicting prognosis of septic patients. We found the initial peak PCT level to be useful as an early predictor of the development of sepsis, severe sepsis/septic shock, and mortality in patients admitted to the ICU. Early and routine serum PCT measurements can help identify patients who would benefit from more intensive treatment, resulting in improved survival among patients admitted to the ICU with sepsis. However, we need more studies, if possible, prospective, randomized controlled, multicenter, open-label intervention trials, to evaluate the effect of PCT-guided decision-making on the clinical outcomes in the ICU setting.
